# Prevalence of hepatitis B virus genotypes among patients with liver disease in Eritrea

**DOI:** 10.1038/s41598-021-90836-w

**Published:** 2021-05-31

**Authors:** Mohammed Elfatih Hamida, Saud Mohammed Raja, Yodahi Petros, Munir Wahab, Yemane Seyoum, Isam Mohammed Elkhidir, Freweini Tekle

**Affiliations:** 1 Department of Microbiology, Orotta College of Medicine and Health Sciences, PO Box: 1034, Asmara, Eritrea; 2 Department of Internal Medicine, Orotta College of Medicine and Health Sciences, Asmara, Eritrea; 3 Unit of Molecular Biology, National Animal and Plant Health Laboratory, Asmara, Eritrea; 4 Department of Microbiology, Faculty of Medicine, University of Khartoum, Khartoum, Sudan; 5 National Health Laboratory, Department of Serology, Ministry of Health, Asmara, Eritrea

**Keywords:** Microbiology, Molecular biology, Gastroenterology, Medical research

## Abstract

Eritrea is an East African multiethnic country with an intermediate endemicity for hepatitis B. Our aim was to establish the most prevalent genotypes of hepatitis B virus (HBV) among patients with liver disease. A total of 293 Eritrean patients with liver disease who were hepatitis B surface antigen (HBsAg) positive were enrolled. All sera were tested for liver transaminases, HBV DNA viral load, and hepatitis B seromarkers including HBsAg, anti-HBcAb (total), HBeAg, and anti-HBeAb. Those reactive for HBsAg and anti-HBc (total) were further tested for HBV genotyping. The median (interquartile range) of HBV DNA viral load and ALT levels were 3.47 (1.66) log IU/mL and 28 (15.3) IU/L, respectively. Using type-specific primer-based genotyping method, 122/293 (41.6%) could be genotyped. Irrespective of mode of occurrence, HBV genotype D (21.3%) was the predominant circulating genotype, followed by genotypes C (17.2%), E (15.6%), C/D (13.1%), and C/E (10.7%). Genotypes C/D/E (7.4%), A/D (4.9%), D/E (4.1%), A (2.5%), and B, A/E, B/E, and A/D/C (0.8%) were also present. HBV in Eritrea is comprised of a mixture of HBV genotypes. This is the first study of HBV genotyping among patients with liver disease in Eritrea.

## Introduction

Hepatitis B virus (HBV) is a partially double-stranded DNA virus belonging to the family Hepadnaviridae^1^. HBV infection is the most frequent cause of liver cirrhosis and hepatocellular carcinoma (HCC) and a cause of chronic infection in more than 240 million individuals worldwide, including 65 million people in Africa^1,2^. Eritrea is an East African multiethnic country with an intermediate HBV seroprevalence as indicated by HBsAg positivity rates of 2.6–3.2%^3^. Ten genotypes of HBV (A–J) with distinct geographic distributions have been recognized^4–6^. Differences in function and structure among genotypes can influence the severity and clinical outcomes of HBV infection as well as complications associated with differences in response to antiviral therapy^7^. Infection by HBV genotypes A and D is more likely to progress to the chronic phase than infection by genotypes B and C, whereas genotypes A and B have higher rates of HBeAg seroconversion than genotypes C and D^8^. Recent studies observed unusual mixed-genotype HBV infections, suggesting overlapping clinical outcomes^9,10^. Genotypes A, D, and E circulate in diverse geographical locales in Africa. Genotype A is the dominant genotype in southern, eastern, and central Africa, genotype D prevails in northern Africa, and genotype E predominates in western Africa^11^.

To the best of our knowledge, no prior study of HBV genotypes has been conducted in Eritrea. Considering Eritrea’s proximity to highly endemic countries where diverse genotypes have been reported including Sudan^10,12,13^, Ethiopia^14^, and Kenya^15^, establishment of the nature of HBV infection in the country becomes of paramount importance. Therefore, the present work aimed to define the most prevalent HBV genotypes among patients with liver disease in Eritrea.

## Methods

### Study design and patients

This was a cross-sectional, laboratory-based study. This study was approved by the ethics committee of the Orotta College of Medicine (Rf. No.: 0041/08/206) and the health facility management division of the Ministry of Health (Approval Number: 15/41/554/17). All patients provided written informed consent to participate in this study. All study procedures were carried out in accordance with the principles of Declaration of Helsinki.

Sera were collected from 293 patients chronically infected with HBV who attended hepatology units and outpatient clinics at Orotta National Referral and Teaching Hospital, Halibet Hospital, Sembel Hospital, and the National Health Laboratory. These are the largest hospitals with highest available diagnostic services in the country. These facilities serve as referral centers to patients coming from different geographical regions of Eritrea, which is divided into six administrative regions (Zobas): Maekel, Debub, Anseba, Gash Barka, Semienawi Keiyh Bahri (SKB), and Debubawi Keiyh Bahri (DKB). The samples were collected between January 2017 and February 2019.

All HBV positive liver disease adult patients who presented to the hospitals for management between January 2017 and February 2019 participated in the study. Patients with acute hepatitis and blood donors who tested positive for HBV surface antigen (HBsAg) were excluded from this study. The patients’ sera were collected and stored at − 20 °C until the experiment was completed.

### Biochemical assays

Levels of liver transaminases (alanine transaminase (ALT) and aspartate transaminase^16^) were measured using an automated clinical chemistry analyzer (Beckman coulter au480, USA) following the manufacturer’s instructions. The upper limit of normal values for both ALT and AST were 40 IU/L^13^.

### Serological evaluation

Sera samples from all patients were re-tested for HBsAg and screened for anti-hepatitis B core antibody (anti-HBc total). Those with detectable anti-HBc total and HBsAg levels were tested for the hepatitis B ‘e’ antigen (HBeAg) and the antibody to HBeAg (anti-HBe) using a commercial enzyme-linked immunosorbent assay (ELISA) kit (Fortress Diagnostics, UK), according to the procedures described by the manufacturers of the Anthos Labtec ELISA 2001 analyzer (Anthos Labtec, Austria).

### HBV viral load quantification

The HBV DNA (viral load) level was assessed by commercial real-time PCR (COBAS AmpliPrep/COBAS TaqMan HBV test, version 2.0, Roche Diagnostics, Germany) for automated specimen processing and amplification, with a lower limit of detection of approximately 20 IU/mL. If the samples had detectable HBV DNA, a quantitative real-time PCR analysis was performed to determine the level of DNA.

### HBV DNA extraction

DNA was extracted from 200 μL of the patients’ sera according to the manufacturer’s instructions using an Analytik Jena DNA mini-extraction kit (Analytik Jena, Germany), eluted with 60 µL of pre-heat RNase-free water, and stored at − 20 °C until use.

### HBV genotype

The HBV DNA genotyping system was based on multiplex-nested PCR using type-specific primers according to that applied by Naito et al.^17^. HBV was detected by amplification of pre-S1 through S genes using universal primers (P1 and S1–2) as outer primers, followed by two different mixtures containing type-specific inner primers, for detecting HBV genotypes according to the previously described method. The PCR primers used in this study are shown in Table 1.

**Table 1:** Primer sequences used for HBV genotyping in this study.

Primers	Sequences^a^	(Specificity, polarity, and position)	Amplicon size (bp)
**First round of PCR**			
P1	5′-TCA CCA TAT TCT TGG GAA CAA GA-3′	**(nt 2823–2845, universal, sense)**	**1063**
S1–2	5′-CGA ACC ACT GAA CAA ATG GC-3′	**(nt 685–704, universal, antisense)**	
**Second round of PCR**			
Mix A			
B2	5′-GGC TCM AGT TCM GGA ACA GT-3′	**(nt 67–86, types A to E specific, sense)**	
BA1R	5′-CTC GCG GAG ATT GAC GAG ATG T-3′	**(nt 113–134, type A specific, antisense)**	**68**
BB1R	5′-CAG GTT GGT GAG TGA CTG GAG A-3′	**(nt 324–345, type B specific, antisense)**	**281**
BC1R	5′-GGT CCT AGG AAT CCT GAT GTT G-3′	**(nt 165–186, type C specific, antisense)**	**122**
Mix B			
BD1	5′-GCC AAC AAG GTA GGA GCT-3′	**(nt 2979–2996, type D specific, sense)**	**119**
BE1	5′-CAC CAG AAA TCC AGA TTG GGA CCA-3′	**(nt 2955–2978, type E specific, sense)**	**167**
BF1	5′-GYT ACG GTC CAG GGT TAC CA-3′	**(nt 3032–3051, type F specific, sense)**	**97**
B2R	5′-GGA GGC GGA TYT GCT GGC AA-3′	**(nt 3078–3097, types D to F specific, antisense)**	

^a^An “M” indicates a nucleotide that may be either A or C; a “Y” indicates a nucleotide that may be either C or T nt, nucleotide.

The total volume of the reaction mixture was 20 µL, which was comprised of 16 µL of nuclease-free water, 1 µL (10 pmol) each of the P1 and S1–2 primers, and 2 µL of the DNA sample, all of which were added to the tube of the ready mix containing 2.5 mM dNTP mix, 2.5 U Taq DNA Polymerase, 1× reaction buffer, and 1× gel loading buffer (iNtRON, Biotechnology, Korea). The mixture was centrifuged at 5000 rpm for 15 s.

The first-round PCR was performed using a master cycler gradient (Ependorf, Germany) by incubating the samples at 94 °C for 5 min, followed by 40 cycles consisting of denaturation at 94 °C for 1 min, annealing at 60 °C for 1 min, and elongation at 72 °C for 2 min. The final elongation step was performed at 72 °C for 5 min.

Two second-round PCRs were performed for each first-round PCR product. Mix A was applied for the identification of genotypes A, B, and C and mix B was applied for the identification of genotypes D, E, and F. Three-microliter aliquots of the first-round PCR product were added to mix A and mix B. The reaction mixture of mix A (iNtRON, Biotechnology, Korea) contained 15 µL of nuclease-free water and 2 µL (10 pmol) of mix A primers whereas the mix B reaction mixture contained 15 µL of nuclease-free water and 2 µL (10 pmol) of mix B primers.

The second-round PCRs underwent 40 cycles with the following parameters: preheating at 95 °C for 5 min, 20 cycles of amplification at 95 °C for 20 s, 60 °C for 20 s, and 72 °C for 30 s, and an additional 20 cycles of 95 °C for 20 s, 62 °C for 20 s, and 72 °C for 30 s. The PCR products were identified by electrophoresis on a 1.5% agarose gel and stained with gel red (Sigma-Aldrich, USA) (1 h and 10 min at 100 V). The bands were evaluated under a UV light transilluminator (UVP, UK). The size of the product bands was estimated according to the migration pattern of the DNA ladder. The expected band sizes were as follows: a genotype of mix A as A (68 bp), B (281 bp), C (122 bp), and mix B genotype as D (119 bp), E (167 bp), and F (97 bp).

### Ethics declarations

This study was approved by the ethics committee of the Orotta College of Medicine and Health Sciences Approval Number (Rf. No.: 0041/08/206) and the health facility management division of the Ministry of Health (Approval Number: 15/41/554/17).

### Consent to participate

All patients provided written informed consent to participate in this study.

### Consent to publish

This article does not reveal any identifiable participant’s data and privacy rights for all participants are observed. Hence, consent for publication is not applicable in this case.

## Results

### Sociodemographic characteristics

Of the 293 patients enrolled in this study, 213 (72.7%) were males and 80 (27.3%) were females. The mean age ± standard deviation (SD) was 41.66 ± 13.84 years with a range of 16 to 78 years. Most of the patients were married (71.7%), followed by single (24.6%) and divorced (3.8%) patients. All administrative regions of Eritrea were represented, with 49.8% of participants from Zoba Maekel, the central region where a large proportion of the Eritrean population resides and where the study was conducted, 21.5% from Debub, 14.3% from Gash Barka, 5.5% from Anseba, 5.5% from SKB, and 3.4% from DKB. Therefore, this study was virtually representative of the population distribution across Eritrea (Table 2).

**Table 2:** Sociodemographic characteristics of patients with liver disease (n = 293).

Variable	Sex^a^		Total^b^ n (%)
	Male n (%)	Female n (%)	
**Age (M = 41.66 ± SD = 13.84, Md = 41.0 ± IQR = 62, Min = 16, Max = 78)**			
Less than 28	42 (71.2)	17 (28.8)	59 (20.1)
29 to 37	44 (69.8)	19 (30.2)	63 (21.5)
38 to 44	34 (64.2)	19 (35.8)	53 (18.1)
45 to 54	43 (74.1)	15 (25.9)	58 (19.8)
55 or above	50 (83.3)	10 (16.7)	60 (20.5)
**Occupation**			
Government employee	92 (80.0)	23 (20.0)	115 (39.2)
Unemployed	34 (44.7)	42 (55.3)	76 (25.9)
Private workers	74 (89.2)	9 (10.8)	83 (28.3)
Students	13 (68.4)	6 (31.6)	19 (6.5)
**Marital status**			
Single	56 (77.8)	16 (22.2)	72 (24.6)
Married	152 (72.4)	58 (27.6)	210 (71.7)
Divorced	5 (45.5)	6 (54.5)	11 (3.8)
**Religion**			
Muslim	28 (75.7)	9 (24.3)	37 (12.6)
Christian	185 (72.3)	71 (27.7)	256 (87.4)
**Zoba**			
Anseba	10 (62.5)	6 (37.5)	16 (5.5)
Debub	42 (66.7)	21 (33.3)	63 (21.5)
Gash Barka	32 (76.2)	10 (23.8)	42 (14.3)
Maekel	107 (73.3)	39 (26.7)	146 (49.8)
Semienawi Keiyh Bahri	14 (87.5)	2 (12.5)	16 (5.5)
Debubawi Keiyh Bahri	8 (80.0)	2 (20.0)	10 (3.4)
**Hospital**			
National Health Laboratory	147 (76.2)	46 (23.8)	193 (65.9)
Orotta Hospital	46 (65.7)	24 (34.3)	70 (23.9)
Halibet Hospital	13 (68.4)	6 (31.6)	19 (6.5)
Sembel Hospital	7 (63.6)	4 (36.4)	11 (3.8)
**Total**	**213 (72.7%)**	**80 (27.3%)**	**293 (100%)**

^a^Percentage is computed from the row total.

^b^Percentage is computed from total liver patients.

### HBV serology and viral load

All patients’ sera were HBsAg-positive and anti-HBc-positive; 20 (6.8%) were HBeAg-positive/anti-HBe-negative, 242(82.6%) were HBeAg-negative/anti-HBe-positive, and 31(10.6%) were negative for both HBeAg and anti-HBe.

The median and interquartile range (IQR) serum levels of ALT and AST were 28 (15.3) and 26 (10.3) IU/L, respectively. The serum ALT levels were higher in the HBeAg-negative samples than in the HBeAg-positive samples.

The HBV DNA viral load was quantified in 122/293 (41.6%) samples, and the median (IQR) was 3.47 (1.66) log IU/mL. An undetectable viral load result was obtained in 171 (58.36%) sera samples. The log median viral load in HBeAg-positive sera [4.2 (5.12) log IU/mL] was higher than that in HBeAg-negative sera [3.3 (1.55) log IU/mL].

### Distribution of HBV genotypes among the study population

This study included 293 samples that were tested by the multiplex-nested PCR technique for HBV DNA using type-specific primers. One hundred and twenty-two samples (41.6%) were positive for HBV DNA, 57.38% of the HBV DNA-positive samples were infected with a single HBV genotype, and the remaining 42.62% had mixed-genotype infections. The mean age of the HBV DNA-positive patients was 40.21 (SD = 12.66) years, and males were predominant (73%).

HBV genotype D (n = 26; 21.3%) was found to be the predominant circulating genotype, followed by genotypes C (n = 21; 17.2%), E (n = 19; 15.6%), C/D (n = 16; 13.1%), and C/E (n = 13; 10.7%). Genotypes C/D/E (n = 9; 7.4%), A/D (n = 6; 4.9%), D/E (n = 5; 4.1%), A (n = 3; 2.5%), and B, A/E, B/E, and A/D/C (each with n = 1; 0.8%) were also present (Table 3).

**Table 3:** Summary and distribution of HBV genotypes among liver disease patients with HBV in Eritrea.

HBV genotypes	n (%)	Age (mean ± SD)	Sex	
			Male, n (%)	Female, n (%)
**HBV mono-genotype infection**				
Genotype A	3 (2.5)	33 ± 5.292	1 (33.3)	2 (66.7)
Genotype B	1 (0.8)	–	1 (100)	0
Genotype C	21 (17.2)	39.19 ± 10.736	14 (66.7)	7 (33.3)
Genotype D	26 (21.3)	39.77 ± 13.168	17 (64.4)	9 (34.6)
Genotype E	19 (15.6)	37.42 ± 10.052	12 (63.2)	7 (36.7)
**HBV mixed-genotype infection**				
Genotype A/D	6 (4.9)	32.17 ± 10.815	6 (100)	0 (0.0)
Genotype A/E	1 (0.8)	–	0 (0.0)	1 (3.0)
Genotype B/E	1 (0.8)	–	1 (100)	0 (0.0)
Genotype C/D	16 (13.1)	42.56 ± 11.302	9 (56.3)	7 (21.2)
Genotype C/E	13 (10.7)	42.54 ± 14.339	13 (100)	0 (0.0)
Genotype D/E	5 (4.1)	55.00 ± 11.467	5 (100)	0 (0.0)
Genotype A/D/C	1 (0.8)	–	1 (100)	0 (0.0)
Genotype C/D/E	9 (7.4)	44.11 ± 17.222	9 (100)	0 (0.0)
Total	**122 (100%)**	**40.21 ± 12.660**	**89 (73%)**	**33 (27%)**

(–) Mean and SD cannot be computed because there was only one patient each with genotype B, A/E, B/E, and A/D/C.

### Serological and biochemical characteristics of patients (n = 122) with HBV genotypes

All the HBV serological and biochemical characteristics of the 122 liver patients with genotyped HBV are shown in Table 4. The results of the HBV mono-genotype analysis revealed that in the HBeAg-positive patients infected with a single genotype, only genotype D (25.0%) was found; there were no patients with genotypes A, B, C, or E. The median (IQR) ALT level of genotype D was 30.0 (17.2) IU/L. Moreover, the median (IQR) AST level was 26.0 (7.3) IU/L, and the average of the log median (IQR) viral loads was 3.39 (2.31) IU/mL. Conversely, the results of the mixed-HBV genotype analysis showed that in the HBeAg-positive patients infected with multiple HBV genotypes, genotypes C/D (10%), C/E (10%), C/D/E (5%), A/D (5%), D/E (5%), and A/E (5%) were found, but there were no patients with genotypes B/E or A/D/C. The median (IQR) ALT levels of genotypes C/D and C/E were 34.0 (16.8) and 26.0 (15.5) IU/L, respectively. Moreover, the median (IQR) AST levels of genotypes C/D and C/E were 30.0 (10.5) and 26.0 (9.6) IU/L, respectively, and the averages of the log median (IQR) viral loads were 2.77 (1.21) and 3.59 (0.75) IU/mL, respectively. There was no significant difference between viral load levels in single HBV-genotype vs. mixed-genotype infection.

**Table 4:** Serological and biochemical characteristics of 122 liver patients with genotyped HBV.

HBV genotypes	Serological and biochemical characteristics				Total n (%)
	HBeAg positive (%)	ALT, IU/L median (IQR)	AST, IU/L median (IQR)	Viral load log median IU/mL (IQR)	
**HBV mono-genotype**					
Genotype A	0	22.0 (–)	20.0 (–)	3.48 (–)	3 (1.0)
Genotype B	0	–	–	–	1 (0.3)
Genotype C	0	26.0 (11.0)	30.0(11.5)	3.63 (1.46)	21 (6.9)
Genotype D	5 (25.0)	30.0 (17.2)	26.0 (7.3)	3.38 (2.31)	26 (8.5)
Genotype E	0	29.0 (14.6)	29.0 (20.5)	3.46 (1.97)	19 (6.2)
**HBV mixed genotypes**					
Genotype A/D	1 (5.0)	24.9 (42.9)	21.0 (23.7)	3.59 (3.91)	6 (2.0)
Genotype A/E	1 (5.0)	–	–	–	1 (0.3)
Genotype B/E	0	–	–	–	1 (0.3)
Genotype C/D	2 (10.0)	34.0 (16.8)	30.0 (10.5)	2.77 (1.21)	16 (5.2)
Genotype C/E	2 (10.0)	26.0 (15.5)	26.0 (9.6)	3.59 (0.75)	13 (4.3)
Genotype D/E	1 (5.0)	38.0 (51.2)	29.0 (40.6)	3.51 (2.02)	5 (1.6)
Genotype A/D/C	0	–	–	–	1 (0.3)
Genotype C/D/E	1 (5.0)	29.0 (22)	22.5 (8.6)	4.09 (1.40)	9 (3.0)

(–) Median and IQR cannot be computed because there was only one patient each with genotypes B, A/E, B/E, and A/D/C.

### Distribution of HBV genotypes in the six geographical Zobas

The distribution of different HBV genotypes was described in the different Zobas of Eritrea **(**Table 5). The majority of patients (45.1%) were from Maekel, followed by Debub (20.2%), Gash Barka (18%), Anseba (8.2%), SKB (4.9%), and DKB (3.3%). However, population adjustment of genotype percentages could not be calculated for each Zoba because of the low numbers of patients in some of the genotype categories.

**Table 5:** Distribution of different HBV genotypes in different Zobas of Eritrea.

HBV genotypes	Zoba						N
	Anseba	Debub	Gash Barka	Maekel	*SKB*	*DKB*	
	N = 10; n (%)	N = 25; n (%)	N = 22; n (%)	N = 55; n (%)	N = 6; n (%)	N = 4; n (%)	
**HBV mono-genotype infection**							
Genotype A	0	1 (4)	0	2 (3.6)	0	0	3
Genotype B	0	0	0	1 (1.8)	0	0	1
Genotype C	5 (50)	4 (16)	4 (18.2)	7 (12.7)	1 (16.7)	0	21
Genotype D	1 (10)	4 (16)	6 (27.3)	13 (23.6)	1 (16.7)	1 (25)	26
Genotype E	1 (10)	4 (16)	3 (13.6)	8 (14.5)	1 (16.7)	2 (50)	19
**HBV mixed-genotype infection**							
Genotype A/D	0	1 (4)	0	4 (7.3)	1 (16.7)	0	6
Genotype A/E	0	1 (4)	0	0	0	0	1
Genotype B/E	0	1 (4)	0	0	0	0	1
Genotype C/D	1 (10)	4 (16)	4 (18.2)	7 (12.7)	0	0	16
Genotype C/E	0	1 (4)	3 (13.6)	8 (14.5)	0	1 (25)	13
Genotype D/E	0	1 (4)	1 (4.5)	2 (3.6)	1 (16.7)	0	5
Genotype A/D/C	0	0	1 (4.5)	0	0	0	1
Genotype C/D/E	2 (20)	3 (12)	0	3 (5.5)	1 (16.7)	0	9

Among the mono-HBV genotype infections in Eritrea, genotype D was predominant in Maekel (23.6%) and Gash Barka (27.3%). HBV genotype C was predominant in Anseba (50%), genotype E was predominant in DKB (50%), and HBV genotypes C, D, and E were equally predominant in Debub (16%) and SKB (16.7%) (Table 5).

Among the mixed-HBV genotype infections, genotype C/D was predominant in Gash Barka (18.2%) and Debub (16%) whereas HBV genotype C/E was predominant in Maekel (14.5%) and DKB (25%). Conversely, HBV genotype C/D/E was predominant in Anseba (20%), whereas genotypes D/E and C/D/E were equally predominant in SKB (16.7%) (Table 5).

## Discussion

HBV is a highly infectious disease and a major threat in developing countries. Hence, this study focuses on assessing the prevalence of HBV genotype in Eritrea. This molecular genotyping of HBV was first of its kind in Eritrea using a PCR-based method, and no data about the genotypes and mutants of HBV in patients with liver disease were previously reported.

Different HBV genotyping methods have been developed including sequencing, INNO-LiPA, restriction fragment polymorphism, multiplex PCR, serotyping, oligonucleotide microarray chips, reverse dot blot, restriction fragment mass polymorphism, invader assay, and real-time PCR. However, the sensitivity, specificity, expense, and time requirements differ among these methods^18^. In this study, we focused on six major genotypes (A–F) among our patients using the multiplex-nested PCR technique developed by Naito et al.^17^. This method appears to have higher sensitivity for detecting mixed genotypes, and it is simple and cost-effective for large population studies with a high accuracy rate of 93%^18^.

The genotypes of HBV were examined in 293 patients, and samples were successfully genotyped in 122 (41.6%) patients. The most important finding in our results was that single-genotype HBV infection (57.38%) was more common than mixed-genotype infection (42.62%). This is in concordance with findings from Taiwan, in which single genotype B infection was most common^19^, and with a study in Egypt, which reported that 87% of patients harbored single-genotype infection, most commonly genotype D^20^. On the contrary, studies from Iraq and Nigeria documented that mixed-genotype infections were present in 75% and 82.6% of patients, respectively^21,22^. When mixed and single infections were considered together, we found that genotype D had the highest prevalence, followed by genotypes C, E, C/D, C/E, C/D/E, A/D, D/E, A, B, A/E, B/E, and A/D/C. However, when only single-genotype infections were considered, genotype D was followed by genotypes C, E, A, and B. On the other hand, genotype C/D infection predominated in mixed-genotype HBV infection, followed by genotypes C/E, C/D/E, A/D, D/E, A/E, B/E, and A/D/C.

The identification of five HBV genotypes (A–E) in this study corroborates their higher prevalence in certain geographical regions in Africa^2,4,23^. We found that the genotypes of HBV in Eritrea conform to those described in the region. Our results of this study concur with previous findings in neighboring countries, including the predominance of HBV genotype D in Sudan^12,13^ and Egypt^24^. Globally, mixed-genotype HBV infection has been reported to be predominant in different regions^10^. Eritrea’s geographical location in the horn of Africa with close proximity to Asia through the Red Sea could explain the nature of distribution of HBV genotypes in the country. The observation of genotype C infection alone or together with genotype D, especially in people hailing from coastal areas of Eritrea, may be attributable to frequent migration and contact with people from regions such as the Middle East because of commercial activities. In addition, mixed-genotype infection was noted in patients from different geographical areas of Eritrea, in line with observations of infection by genotypes A, D, and E, including single and mixed infections, in Sudan^10^. In addition, the observation of mixed-genotype infection in this country could be linked to the significant number of refugees returning from neighboring and distant countries^25^.

The clinical impact of HBV genotype D has not been studied extensively. Emerging evidence suggests that patients with genotype D infection may develop fulminant hepatitis at high frequency^26^. The prevalence of different HBV genotypes in our study subjects provides a basis to compare different parameters in a stratified manner for various genotypes (Table 4). The viral loads in patients with single-genotype HBV were similar to that of those with mixed genotypes. This finding may be attributed to the nature of the infection in the region such as predominance of HBV with low viral replication phase, similar to a previous study in the country^3^.

Knowing the predominant HBV genotype in specific areas is important for assessing diagnostic capabilities and vaccine efficacy^1^. Our study narrows the existing gaps in HBV molecular research in Eritrea. All of our isolates were obtained from patients with chronic HBV infection in hospital settings. Generally, patients with liver disease who are identified as reactive for HBV infection during the study were not monitored or evaluated concerning the exact stage of their liver disease such as cirrhosis or HCC. Hence, we suggest that HBV genotyping of patients be studied in correlation with the clinical progression of liver disease to provide a clear clinical picture and molecular epidemiology. Furthermore, future studies are expected to evaluate the clinical relevance, treatment response, and rates of coinfection, which may affect disease outcome.

## Conclusion

In conclusion, this study portrays the overall prevalence of HBV genotypes among Eritrean liver disease patients infected with HBV who seek medical attention at the hospital setting. In mono-HBV genotype infections, genotype D was the most prevalent genotype. In mixed-HBV genotype infections, genotype C/D was the most prevalent among the study region. The high prevalence of genotype D was similar to most of the previous studies, including those in Saudi Arabia^27^. This results in poor outcomes in clinical management. In the future, clinical trials and treatment regimens should be postulated individually based on the genotype to effectively manage chronic HBV infection. To that ends, a prospective nation-wide population study of HBV genotype distribution and clinical outcome is recommended.

## Data Availability

All datasets used for this study are available from the corresponding author on reasonable request.
